# Inhibition of HIV-1 gene expression by Ciclopirox and Deferiprone, drugs that prevent hypusination of eukaryotic initiation factor 5A

**DOI:** 10.1186/1742-4690-6-90

**Published:** 2009-10-13

**Authors:** Mainul Hoque, Hartmut M Hanauske-Abel, Paul Palumbo, Deepti Saxena, Darlene D'Alliessi Gandolfi, Myung Hee Park, Tsafi Pe'ery, Michael B Mathews

**Affiliations:** 1 Department of Biochemistry & Molecular Biology, UMDNJ-New Jersey Medical School, NJ 07103, USA; 2 Department of Obstetrics, Gynecology & Women's Health, UMDNJ-New Jersey Medical School, NJ 07103, USA; 3 Department of Pediatrics, UMDNJ-New Jersey Medical School, NJ 07103, USA; 4 Department of Chemistry, Manhattanville College, NY 10577, USA; 5 National Institute for Dental and Craniofacial Research, NIH, MD 20892, USA; 6 Department of Medicine, UMDNJ-New Jersey Medical School, NJ 07103, USA; 7 Current Address: Section of Infectious Diseases and International Health, Dartmouth Medical Center, One Medical Center Drive, Lebanon, NH 03756, USA

## Abstract

**Background:**

Eukaryotic translation initiation factor eIF5A has been implicated in HIV-1 replication. This protein contains the apparently unique amino acid hypusine that is formed by the post-translational modification of a lysine residue catalyzed by deoxyhypusine synthase and deoxyhypusine hydroxylase (DOHH). DOHH activity is inhibited by two clinically used drugs, the topical fungicide ciclopirox and the systemic medicinal iron chelator deferiprone. Deferiprone has been reported to inhibit HIV-1 replication in tissue culture.

**Results:**

Ciclopirox and deferiprone blocked HIV-1 replication in PBMCs. To examine the underlying mechanisms, we investigated the action of the drugs on eIF5A modification and HIV-1 gene expression in model systems. At early times after drug exposure, both drugs inhibited substrate binding to DOHH and prevented the formation of mature eIF5A. Viral gene expression from HIV-1 molecular clones was suppressed at the RNA level independently of all viral genes. The inhibition was specific for the viral promoter and occurred at the level of HIV-1 transcription initiation. Partial knockdown of eIF5A-1 by siRNA led to inhibition of HIV-1 gene expression that was non-additive with drug action. These data support the importance of eIF5A and hypusine formation in HIV-1 gene expression.

**Conclusion:**

At clinically relevant concentrations, two widely used drugs blocked HIV-1 replication *ex vivo*. They specifically inhibited expression from the HIV-1 promoter at the level of transcription initiation. Both drugs interfered with the hydroxylation step in the hypusine modification of eIF5A. These results have profound implications for the potential therapeutic use of these drugs as antiretrovirals and for the development of optimized analogs.

## Background

Since its discovery in 1981, human immunodeficiency virus type 1 (HIV-1) has led to the death of at least 25 million people worldwide. Although there have been great strides in behavioral prevention and medical treatment of HIV/AIDS, for the last several years the pandemic has claimed about 2.5 million lives annually  and remains unchecked. It is predicted that 20-60 million people will become infected over the next two decades even if there is a 2.5% annual decrease in HIV infections [1]. Studies of the HIV-1 life cycle led to the development of drugs targeting viral proteins important for viral infection, most notably reverse transcriptase and protease inhibitors. Despite the success of combinations of these drugs in highly active antiretroviral therapy (HAART), the emergence of drug-resistant HIV-1 strains that are facilitated by the high mutation and recombination rates of the virus in conjunction with its prolific replication poses a serious limitation to current treatments. An attractive strategy to circumvent this problem entails targeting host factors that are recruited by the virus to complete its life cycle.

HIV-1 replication requires numerous cellular as well as viral factors, creating a large set of novel potential targets for drug therapy [2-4]. The premise is that compounds directed against a cellular factor that is exploited during HIV-1 gene expression may block viral replication without adverse effects. One such cellular factor is eukaryotic initiation factor 5A (eIF5A, formerly eIF-4D). eIF5A is the only protein known to contain the amino acid hypusine. The protein occurs in two isoforms, of which eIF5A-1 is usually the more abundant [5,6], and has been implicated in HIV-1 replication [7]. Over-expression of mutant eIF5A, or interference with hypusine formation, inhibits HIV-1 replication [8-11]. eIF5A has been implicated in Rev-dependent nuclear export of HIV-1 RNA [7,8,10,12-15].

Originally characterized as a protein synthesis initiation factor [16], the precise function(s) of eIF5A remain elusive. It has been implicated in translation elongation [17-19], the nucleo-cytoplasmic transport of mRNA [20], mRNA stability [21], and nonsense-mediated decay (NMD) [22]. It is tightly associated with actively translating ribosomes [17,18,21,23,24] and is an RNA-binding protein [25,26]. Consequently, it has been suggested to function as a specific initiation factor for a subset of mRNAs encoding proteins that participate in cell cycle control [27,28]. Its biological roles encompass cancer, maintenance of the cytoskeletal architecture, neuronal growth and survival, differentiation and regulation of apoptosis [16,29-34]. The mature form of eIF5A-1 is associated with intraepithelial neoplasia of the vulva [35] while the eIF5A-2 gene is amplified and expressed at high level in ovarian carcinoma and cancer cell lines [30,36,37]. Reduction of eIF5A levels slowed proliferation and led to cell cycle arrest in yeast [27,34,38,39]. In mammalian cells, inhibitors of hypusine formation arrest the cell cycle at the G1/S boundary [40-43]; they also led to reduced proliferation of leukemic cells and sensitized Bcr-Abl positive cells to imatinib [44].

**Figure 1:**
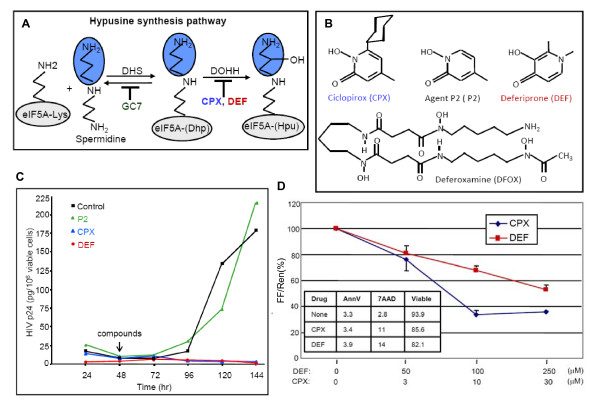
**Inhibition of HIV replication by drugs that block eIF5A modification**. **A**. Hypusination of eIF5A (gray) occurs in two steps: the transfer, catalyzed by DHS, of an aminobutyl moiety (blue) from spermidine onto the side chain of eIF5A lysine-50, yielding deoxyhypusine (Dhp); and its subsequent hydroxylation, catalyzed by DOHH, yielding hypusine (Hpu). DHS is inhibited by GC7 and DOHH by CPX and DEF, as indicated. **B**. Structures of CPX, Agent P2, DEF and DFOX. **C**. CPX and DEF inhibit HIV replication in infected PBMCs. Infected PBMCs that were isolated from a single donor were co-cultured with uninfected PBMCs. CPX (30 μM), P2 (30 μM), or DEF (250 μM) were added 48 hr later. Amount of released p24 protein per million viable cells was determined every 24 hr. **D**. CPX and DEF inhibit gene expression from an HIV molecular clone in a dose dependant manner. The molecular clone pNL4-3-LucE^- ^and pCMV-Ren were transfected into 293T cells and drugs were added to the concentrations shown. Dual luciferase assays were conducted at 12 hr post-transfection. Firefly (FF) luciferase expression was normalized to *Renilla *luciferase (Ren) from pCMV-Ren (mean of 2 experiments in duplicate, ± SD). Inset shows CPX and DEF effects on apoptosis and cell viability in untransfected 293T cultures as measured by staining with annexin V (AnnV) and 7-amino-actinomycin D (7AAD). Data are means of three time points (12, 18 and 24 hr) presented as percentages.

Maturation of eIF5A involves both acetylation and hypusination and is necessary for most if not all of its biological roles [45-48]. Hypusine is formed by the posttranslational modification of a specific lysine residue in both eIF5A isoforms throughout the archaea and eukaryota [49]. Hypusine, the enzymes responsible for its formation, and eIF5A itself, are highly conserved in eukaryotes [31,50,51]. This modification of eIF5A entails two consecutive steps (Fig. 1A). In the first step, deoxyhypusine synthase (DHS) catalyzes the cleavage of the polyamine spermidine and the transfer of its 4-aminobutyl moiety to the ε-amino group of lysine-50 (in human eIF5A-1) of the eIF5A precursor, yielding a deoxyhypusine-containing intermediate. In the second step, deoxyhypusine hydroxylase (DOHH) hydroxylates the deoxyhypusyl-eIF5A intermediate to hypusine-containing mature eIF5A using molecular oxygen [49]. DOHH is essential in *C. elegans *and *D. melanogaster*, but not in *S. cerevisiae *[52,53], indicative of a requirement for fully modified eIF5A at least in higher eukaryotes. The non-heme iron in the catalytic center of DOHH renders the enzyme susceptible to small molecule inhibitors that conform to the steric restrictions imposed by the active site pocket and interact with the metal via bidentate coordination [54].

The pharmaceuticals ciclopirox (CPX) and deferiprone (DEF) are drugs that block DOHH activity [11,41,55]. Both drugs are metal-binding hydroxypyridinones (Fig. 1B). CPX is a topical antifungal (e.g., Batrafen™) and DEF is a medicinal chelator (e.g., Ferriprox™) taken orally for systemic iron overload [56,57]. DEF has been shown to inhibit HIV-1 replication in latently-infected ACH-2 cells after phorbol ester induction [11], and in peripheral blood lymphocytes but not in macrophages [58].

Here we report that clinically relevant concentrations of CPX and DEF block HIV-1 infection of human peripheral blood mononuclear cells (PBMCs). We investigated the early effects of the drugs on gene expression from HIV-1 molecular clones in model systems. Both drugs disrupt eIF5A maturation by blocking the binding of DOHH to its substrate. We show that they inhibit gene expression from HIV molecular clones at the RNA level. The drugs act specifically on the viral LTR, with no discernible requirement for viral proteins, and reduce RNA synthesis from the HIV-1 promoter at the level of transcription initiation. Consistent with eIF5A being a target for these drugs, partial depletion of eIF5A-1 by RNA interference also inhibits HIV-1 promoter-driven gene expression, and this inhibition is non-additive with that caused by the drugs. We conclude that the action of CPX and DEF is at least in part a result of the inhibition of eIF5A hydroxylation, suggesting that cellular DOHH could serve as an antiretroviral target without incurring gross topical or systemic toxicity.

## Results

### Antiviral activity of ciclopirox and deferiprone

To examine the effect of CPX and DEF on HIV-1 propagation, uninfected PBMCs from healthy donors were co-cultured with HIV-infected PBMCs, and virus production was monitored by the p24 capture assay. In untreated cultures, p24 was first detected at 96 hr and its levels increased until up to 144 hr (Fig. 1C; Control). Addition of CPX and DEF at 48 hr, to 30 μM and 250 μM respectively, reduced p24 to baseline levels. This profound inhibition is due, at least in part, to activation of apoptosis at later stages of infection ([11]; unpublished data). These concentrations are within the clinically relevant range and are sufficient to block DOHH activity and eIF5A modification (see below). Agent P2, a chelation homolog of CPX (Fig. 1B), did not impede p24 production (Fig. 1C). These findings suggested that the inhibition of HIV replication by CPX and DEF could be due to inhibition of DOHH and eIF5A maturation.

We selected 293T cells as a model system to explore the relationship between the drugs, eIF5A, and HIV gene expression. These cells efficiently transcribe HIV-1 genes from molecular clones as well as subviral constructs, allowing for early detection of changes in HIV gene expression. To establish the system, we examined the effect of CPX and DEF on the expression of firefly luciferase (FF) from the HIV-1 molecular clone pNL4-3-LucE^- ^that was engineered to carry the FF gene in place of the viral *nef *gene. The molecular clone was transfected into 293T cells together with the pCMV-Ren vector that expresses *Renilla *luciferase (Ren) from the cytomegalovirus (CMV) immediate early promoter as a control for transfection efficiency and non-specific effects of the compounds. Dual luciferase assays were conducted at 12 hr post-transfection. Results are expressed as relative luciferase activity (FF:Ren). As shown in Figure 1D, the drugs repressed expression from the HIV-1 molecular clone in a dose dependent fashion. Long-term drug exposure leads to pleiotropic effects including apoptosis ([11]; unpublished data), but marginal 293T cell death was observed within 24 hr using these concentrations of CPX and DEF (Fig. 1D, inset). We therefore characterized the action of CPX and DEF on eIF5A and HIV gene expression in 293T cells during the first 12 to 24 hr of drug treatment.

### Drug effects on eIF5A and DOHH

**Figure 2:**
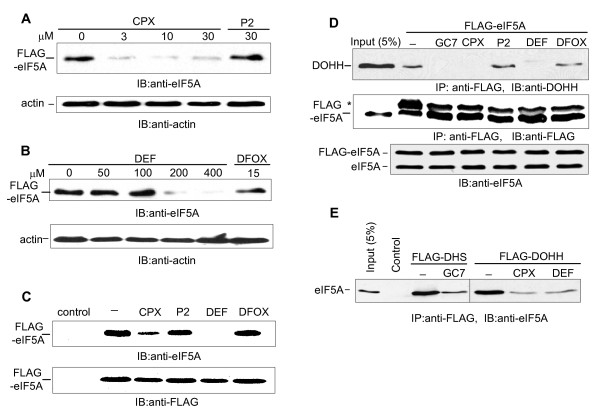
**Ciclopirox and deferiprone prevent the maturation of eIF5A**. **A**. Drug inhibition of eIF5A modification in 293T cells. Cells transfected with FLAG-tagged eIF5A were untreated or treated with increasing concentrations of CPX as indicated, or with agent P2. At 24 hr post-transfection, whole cell extract (WCE) was analyzed by immunoblotting with the NIH-353 anti-eIF5A antibody (upper panel) and anti-actin antibody (lower panel). **B**. Cells transfected with FLAG-tagged eIF5A were untreated or treated with increasing concentrations of DEF as indicated, or with DFOX. Cells were processed as in A. **C**. Cells transfected with FLAG-tagged eIF5A were treated with CPX (30 μM), P2 (30 μM), DEF (250 μM), DFOX (10 μM), or no drug (-). At 24 hr post-transfection, WCE was analyzed by immunoblotting with the NIH-353 anti-eIF5A antibody (upper panel) and anti-FLAG antibody (lower panel). The control culture was transfected with empty vector and no drug was added. **D**. Inhibition of enzyme-substrate binding. 293T cells transfected with FLAG-eIF5A were untreated (-) or treated with GC7 (10 μM) or CPX (30 μM), P2 (30 μM), DEF (250 μM), or DFOX (10 μM). WCE prepared at 24 hr post-transfection was immunoprecipitated with anti-FLAG antibody. Immunoprecipitates were immunoblotted with antibodies against DOHH (top panel) and FLAG (bottom panel). (*)-IgG light chain. **E**. 293T cells transfected with FLAG-DHS, FLAG-DOHH or empty vector (Control) were treated with GC7, CPX, or DEF, or no drug (-) at the same concentration as in panel D. Immunoprecipitates obtained with anti-FLAG antibody were immunoblotted and probed with anti-eIF5A antibody (BD). Input: WCE equivalent to 5% of the input was immunoblotted as a further control.

To examine the effect of the drugs on the synthesis of modified eIF5A, 293T cells transfected with a FLAG-tagged eIF5A expression vector were simultaneously treated with CPX or DEF. FLAG-eIF5A was monitored using NIH-353 and anti-FLAG antibodies (Fig. 2A,B). The NIH-353 antibody reacts preferentially with post-translationally modified eIF5A [35]. CPX reduced the appearance of mature eIF5A over the 3-30 μM concentration range, while DEF was effective at 200-400 μM. The drugs did not alter the expression of actin. Comparable results have been obtained in other cell types by spermidine labeling of eIF5A [41]. In addition to the CPX homolog Agent P2, we used deferoxamine (DFOX; Desferal™) as a control compound. DFOX, a metal-binding hydroxamate like CPX and Agent P2 (Fig. 1B), is a globally used medicinal iron chelator [59] that does not inhibit HIV-1 infection [60]. In contrast to CPX and DEF, P2 and DFOX had little or no effect on the appearance of mature FLAG-eIF5A (Fig. 2A,B), indicating that the ability to chelate iron is insufficient to inhibit DOHH and the maturation of eIF5A. None of these compounds reduced the overall expression of the FLAG-eIF5A protein detectably (Fig. 2C), ruling out general inhibitory effects on gene expression. Based on these results, we used 30 μM CPX and 250 μM DEF for subsequent experiments.

eIF5A forms tight complexes with its modifying enzymes. Unmodified eIF5A (lysine-50) immunoprecipitates with DHS [61,62], and deoxyhypusyl-eIF5A interacts with DOHH *in vitro *[63]. We discovered that the deoxyhypusyl-eIF5A:DOHH complex formed *in vivo *can be detected by immunoprecipitation from cell extracts. Taking advantage of this finding, we tested the effects of the drugs on the enzyme-substrate interaction. FLAG-eIF5A was expressed in 293T cells. Complexes that immunoprecipitated with anti-FLAG antibody were immunoblotted and probed with antibodies against DOHH. Endogenous DOHH co-immunoprecipitated with FLAG-eIF5A, and this association was largely prevented by treatment with CPX or DEF (Fig. 2D, top panel). Consistent with their inability to inhibit eIF5A maturation, neither P2 or DFOX prevented the formation of the eIF5A:DOHH complex. As a further control, we included the DHS inhibitor GC7 [64,65] in this assay. No DOHH was associated with FLAG-eIF5A in the presence of GC7 because it prevents the synthesis of deoxyhypusyl-eIF5A. As expected, none of the compounds affected the immunoprecipitation of FLAG-eIF5A (Fig. 2D, middle panel) or the expression of endogenous eIF5A (Fig. 2D, bottom panel). Reciprocally, the interaction between endogenous eIF5A and tagged DOHH was inhibited by CPX and DEF (Fig. 2E, right). Similarly, the interaction of endogenous eIF5A with tagged DHS was inhibited by GC7 (Fig. 2E, left) but was resistant to CPX and DEF (not shown). We conclude that CPX and DEF, but not P2 or DFOX, target DOHH and inhibit its interaction with its substrate, deoxyhypusyl-eIF5A.

### Inhibition of gene expression from HIV-1 molecular clones

**Figure 3:**
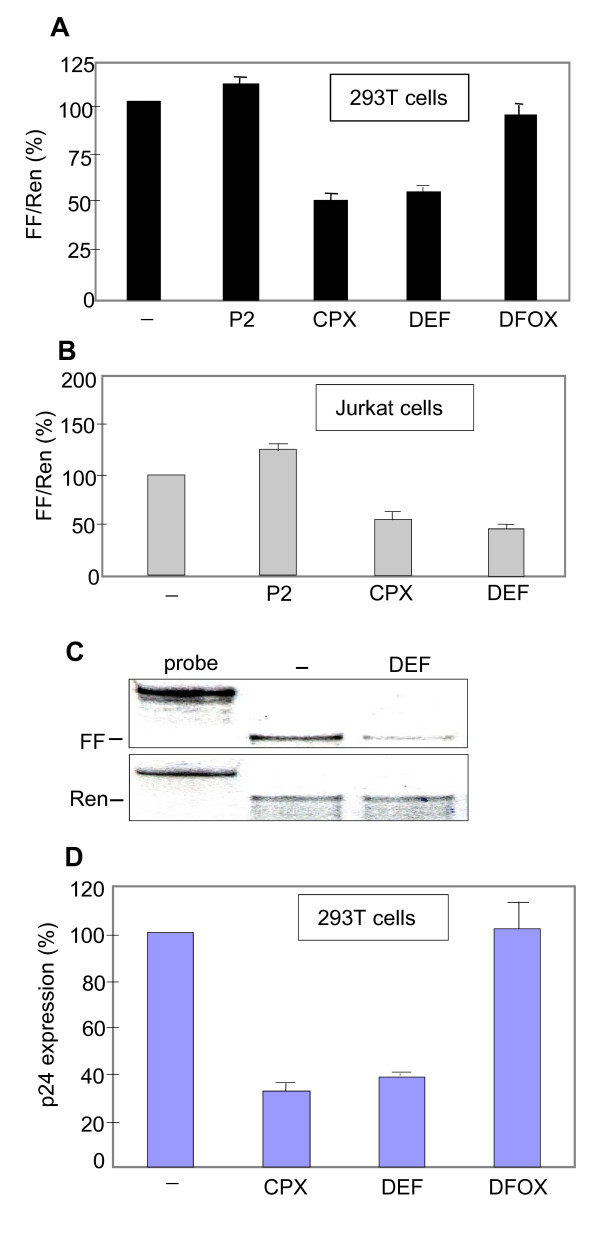
**Drug effects on luciferase expression from an HIV-1 molecular clone**. **A**. Comparison of drug effects on luciferase expression from the pNL4-3-LucE^- ^molecular clone in 293T cells. The molecular clone pNL4-3-LucE^- ^and pCMV-Ren were transfected into 293T cells. Drugs were added where indicated at the following concentrations: P2 (30 μM), CPX (30 μM), DEF (250 μM), or DFOX (15 μM). Dual luciferase assays were conducted at 12 hr post-transfection. Firefly (FF) luciferase expression was normalized to *Renilla *luciferase (Ren) from pCMV-Ren (mean of 2 experiments in duplicate, ± SD). **B**. Expression in Jurkat cells was assayed essentially as in panel A. **C**. Firefly and *Renilla *luciferase RNA expression was analyzed in 293T cells treated as in panel A by RPA using ^32^P- [UTP] labeled antisense RNA probes corresponding to the C-termini of the FF and Ren luciferase mRNAs. **D**. Comparison of drug effects on p24 expression from the pNL4-3-LucE^- ^molecular clone in 293T cells. Drugs were added where indicated to the same concentrations as in A. p24 levels were determined in cell extract at 12 hr post-transfection.

To explore the mechanism whereby CPX and DEF inhibit HIV gene expression, we first examined the specificity of their effect on the expression from the pNL4-3-LucE^- ^molecular clone. Exposure to CPX and DEF repressed expression from the HIV-1 molecular clone by ~50%, as shown above (Fig. 1D), whereas P2 and DFOX were ineffective (Fig. 3A). The drugs had no effect on CMV-driven *Renilla *luciferase expression. Similar results were obtained in transfected Jurkat T cells (Fig. 3B). RNase protection assays (RPA) showed that the inhibition of luciferase activity by DEF (Fig. 3C) or CPX (not shown) was reflected in decreased accumulation of FF mRNA, while no change was observed in the accumulation of Ren mRNA from the CMV promoter. Thus, the drugs specifically inhibited luciferase expression from the HIV-1 molecular clone at the RNA level.

**Figure 4:**
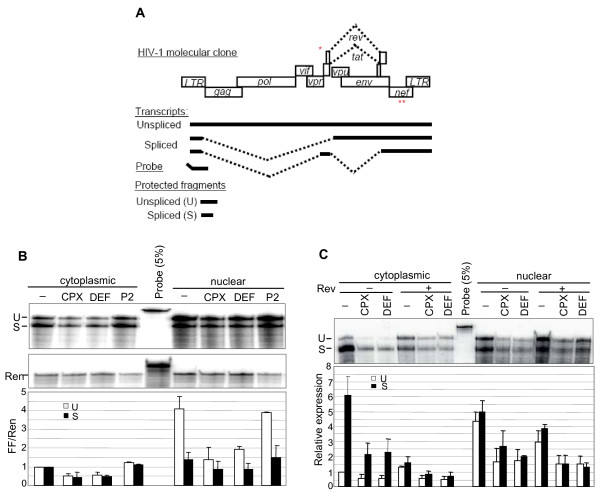
**Inhibition of HIV RNA expression from molecular clones**. **A**. Schematic of HIV-1 provirus showing major transcripts, the position of the antisense probe, and fragments protected by RPA from spliced (S) and unspliced (U) transcripts. The positions of the Rev start codon mutation in pMRev(-) and the FF substitution in pNL4-3-LucE^- ^are marked with one and two asterisks, respectively. **B**. Cytoplasmic and nuclear RNA isolated at 12 hr from 293T cells co-transfected with pNL4-3-LucE^- ^and pCMV-Ren. Drugs were added where indicated at concentrations specified in Fig. 2D. RNA was isolated at 12 hr post-transfection. Autoradiograms display RPA fragments corresponding to HIV and *Renilla *RNAs (upper and middle panels, respectively). *Renilla *RNA was analyzed as in Fig. 3. The lower panel displays quantitation of protected spliced and unspliced RNA fragments relative to the *Renilla *RNA fragment (mean of 2 experiments in duplicate, ± SD). Probe: undigested probe in an amount equivalent to 5% of the input to the protection assays was run as a control. **C**. Effect of Rev. RNA from 293T cells transfected with the Rev-defective HIV molecular clone pMRev(-) together with (+) or without (-) Rev expression vector. RNA was isolated at 15 hr post-transfection. The lower panel displays quantitation of protected spliced and unspliced RNA fragments relative to the cytoplasmic unspliced control RNA (mean of 2 experiments in duplicate, ± SD).

Both CPX and DEF also inhibited HIV p24 expression from the molecular clone by ~60%, whereas DFOX had no effect (Fig. 3D). We next examined the effects of CPX and DEF on viral mRNA expression. The sensitivity of FF expression from pNL4-3-LucE^- ^to these drugs suggested that the inhibition of RNA accumulation is independent of Rev since the FF sequences are substituted into the *nef *gene which gives rise to spliced mRNA. To determine whether the action of CPX and DEF is exerted at the level of the accumulation, splicing or nucleo-cytoplasmic distribution of HIV RNA, we transfected pNL4-3-LucE^- ^into 293T cells and monitored spliced and unspliced HIV RNA after drug treatment. RNase protection assays were carried out using a probe complementary to the 5' region of all HIV-1 transcripts [66]. The probe spans the major splice donor site so as to generate two sizes of protected fragments: unspliced RNA protects an RNA fragment 50 nucleotides (nt) longer than that from spliced RNAs (Fig. 4A). CPX and DEF, but not P2, reduced the level of both spliced and unspliced RNAs by ~50% (Fig. 4B). A similar reduction was observed in both the cytoplasmic and nuclear fractions. In contrast, the production of *Renilla *luciferase RNA driven by the CMV promoter was unchanged in the nucleus and cytoplasm after drug treatment (Fig. 4B). Thus, the drugs cause an overall inhibition in HIV RNA expression as early as 12 hr after drug addition.

These experiments did not disclose a significant effect on the splicing or export of viral RNA as a result of treatment with CPX or DEF. Because previous reports indicated that modified eIF5A is involved in the Rev-dependent export of unspliced and underspliced HIV-1 RNAs [7,10,13], we examined whether the drugs affect the splicing or export of viral RNAs mediated by Rev. The *rev*-defective molecular clone pMRev(-) contains the entire HIV-1 genome but Rev expression is prevented by substitutions in its initiation codon [67]. To compare the inhibitory effect of CPX and DEF in the presence and absence of Rev, cells were transfected with pMRev(-), either with or without a Rev expression vector, and RNA was analyzed by RPA as above. As expected, in the absence of Rev there was very little unspliced RNA in the cytoplasm although substantial levels were present in the nucleus, and Rev expression increased the level of unspliced RNA in the cytoplasm (Fig. 4C). Treatment with CPX or DEF reduced the levels of both spliced and unspliced RNAs in the nucleus and cytoplasm by 2-3 fold irrespective of the presence or absence of Rev (Fig. 4C). Similar data were obtained in COS7 cells (not shown). These results indicate that the drugs inhibited HIV-1 RNA accumulation by a mechanism that is independent of Rev-mediated viral RNA splicing and export. This finding is consistent with the inhibition of FF expression from pNL4-3-LucE^- ^(Fig. 3). Furthermore, since pMRev(-) contains an intact *nef *gene, we can rule out the possibility that the findings with pNL4-3lucE^- ^are a consequence of the absence of *nef *from this molecular clone.

### Genetic requirements for drug sensitivity

**Figure 5:**
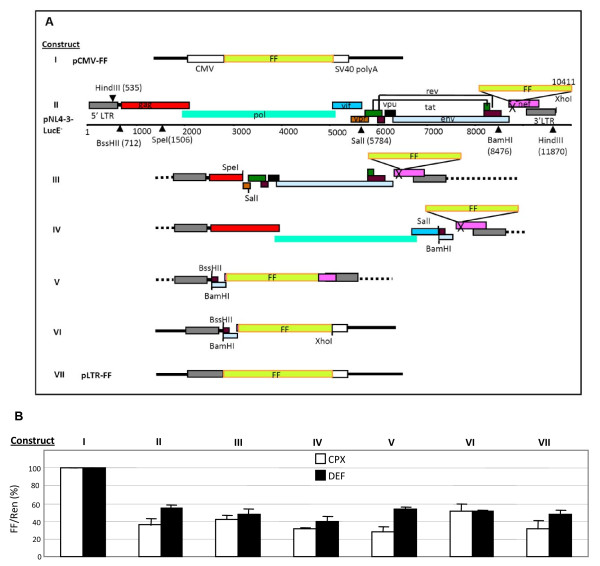
**Sequence requirements for the drug sensitivity of the HIV molecular clone**. **A**. Schematic of constructs expressing firefly luciferase from the CMV promoter (construct I, pCMV-FF) or the HIV promoter. Constructs III, IV and V were generated by deleting sequences from pNL4-3-LucE^- ^(construct II). Construct VI was made by replacing the 3'LTR in construct V with the SV40 poly(A) sequence from pGL2TAR. Construct VII is a chimera of pGL2TAR and construct VI. **B**. CPX and DEF sensitivity of the constructs. Firefly luciferase expression from each construct was normalized to *Renilla *luciferase expression from pCMV-Ren as in Fig. 3, and presented as a percentage of the control ratio obtained in the absence of drugs.

The data obtained with pMRev(-) excluded involvement in the drug responses of the *env *mutation, *nef *deletion and FF gene insertion in pNL4-3lucE^-^, as well as the *rev *gene. To search for viral elements that confer sensitivity to CPX and DEF in these short-term experiments, we generated a series of truncations of the HIV-1 genome. Unique restriction sites were exploited to delete major open reading frames from pNL4-3-lucE^- ^(Fig. 5A). Compared to the parental clone (construct II), construct III has a deletion of nt 1506-5784 affecting *gag*, *pol *and *vif*, while construct IV lacks nt 5784 - 8476 eliminating the expression of *vpr*, *vpu*, *tat*, *rev *and *env*. These two deletions encompass nearly all of the viral coding sequences. Nevertheless, FF expression from these constructs was inhibited ≥50% by CPX and DEF within 12 hr (Fig. 5B). (Note that Tat-deficient constructs were complemented by co-transfection of a Tat expression vector in these assays.) Subsequently, we produced construct V by deleting all the open reading frames except for luciferase from the *nef *coding region. Drug inhibition of this construct, which retains only ~1,967 nt of viral sequence, was also ≥50% (Fig. 5).

All of these constructs have two intact LTRs, derived from the 5' and 3' ends of the molecular clone. When the 3'-LTR of construct V, which contains the HIV-1 poly(A) signal, was replaced by a poly(A) signal from SV40 in construct VI, expression was still inhibited ~50% by CPX and DEF (Fig. 5) indicating that the 3'-LTR is not the determining feature. Construct VI contains 321 nt of *env *as well as the *nef *ATG, but these sequences can also be excluded as demonstrated by construct VII (pLTR-FF) in which the 5' LTR is the only segment derived from HIV (Fig. 5). By contrast, expression from pCMV-FF (construct I) was unaffected by CPX and DEF (Fig. 5), consistent with our findings with pCMV-Ren (Figs. 3 and 4). Thus, the inhibition of gene expression by both drugs is specific for the HIV 5'-LTR.

### CPX and DEF inhibit transcription initiation at the HIV-1 promoter

**Figure 6:**
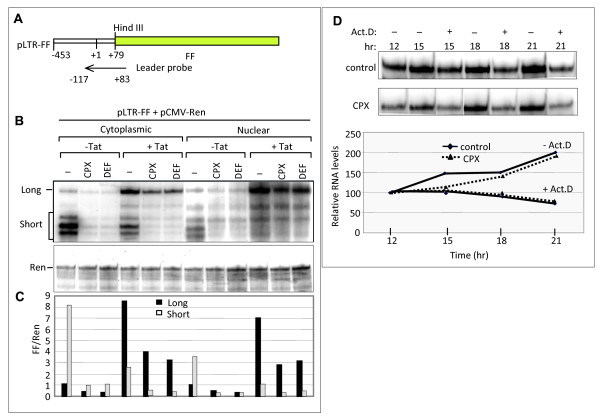
**Inhibition of gene expression by CPX and DEF is promoter specific**. **A-C**. Inhibition is independent of Tat. Total RNA was isolated 15 hr after transfection with pLTR-FF and pCMV-Ren in the absence or presence of Tat expression plasmid. Drugs were added as in Fig. 2D. RPA analysis was conducted by probing with antisense HIV-1 leader RNA probe complementary to LTR nt +83 to -117 (panel A). Protected fragments corresponding to promoter-proximal (Short) and promoter-distal (Long) transcripts were resolved (panel B) and quantified relative to *Renilla *RNA (panel C) analyzed as in Fig. 3. **D**. Stability of RNA transcribed from the HIV promoter in the presence of CPX. Actinomycin D (1 μg/ml) was added at 12 hr where indicated. RPA was carried out for FF mRNA as in Fig. 3. Upper panels: expression of FF RNA from the HIV promoter in control and CPX treated cells. Lower panel: FF mRNA decay rate in the presence or absence of CPX plotted relative to levels at 12 hr post-transfection (~50% less in the presence of CPX).

Results of the deletion analysis implied that sensitivity to the drugs is conferred by the promoter or another feature in the HIV-1 LTR. A conspicuous feature of HIV transcription is its dependence on the viral Tat protein and the cellular complex P-TEFb (positive transcription elongation factor b) that cooperate to ensure processive transcription and the formation of long viral transcripts [68]. To determine whether the drugs inhibit at the elongation step, we examined their effect on HIV-1 transcripts generated in COS7 cells co-transfected with pLTR-FF and pCMV-Ren in the presence or absence of a Tat expression vector. Nuclear and cytoplasmic RNA was analyzed in RNase protection assays using a probe complementary to the promoter-proximal region of HIV transcripts (Fig. 6A). As expected, short fragments corresponding to RNA of ~55-59 nt predominated in the absence of Tat, whereas longer fragments of ~83 nt accumulated in its presence (Fig. 6B) [69,70]. Similar observations were made in the cytoplasm and nucleus. Treatment with CPX and DEF diminished both signals by 50-80% irrespective of the presence or absence of Tat (Fig. 6B,C). These results argue against a specific effect at the level of HIV transcription elongation.

To examine the possibility that the drugs decrease the stability of RNA transcribed from the HIV promoter, cells transfected with pLTR-FF were incubated in the presence or absence of CPX. Actinomycin D was added to some cultures 12 hr later to block further transcription, and FF RNA was monitored by RPA at intervals thereafter (Fig. 6D, top panel). FF RNA levels were quantified and normalized to the levels at 12 hr (Fig. 6D, bottom panel). As expected, FF RNA continued to accumulate in the absence of actinomycin D but declined in its presence. The rate of RNA decay was not affected by the presence of CPX (Fig. 6D). Similar results were obtained with DEF (data not shown). We therefore conclude that the drugs inhibit HIV-1 transcription initiation.

### Inhibition of eIF5A production reduces HIV gene expression

**Figure 7:**
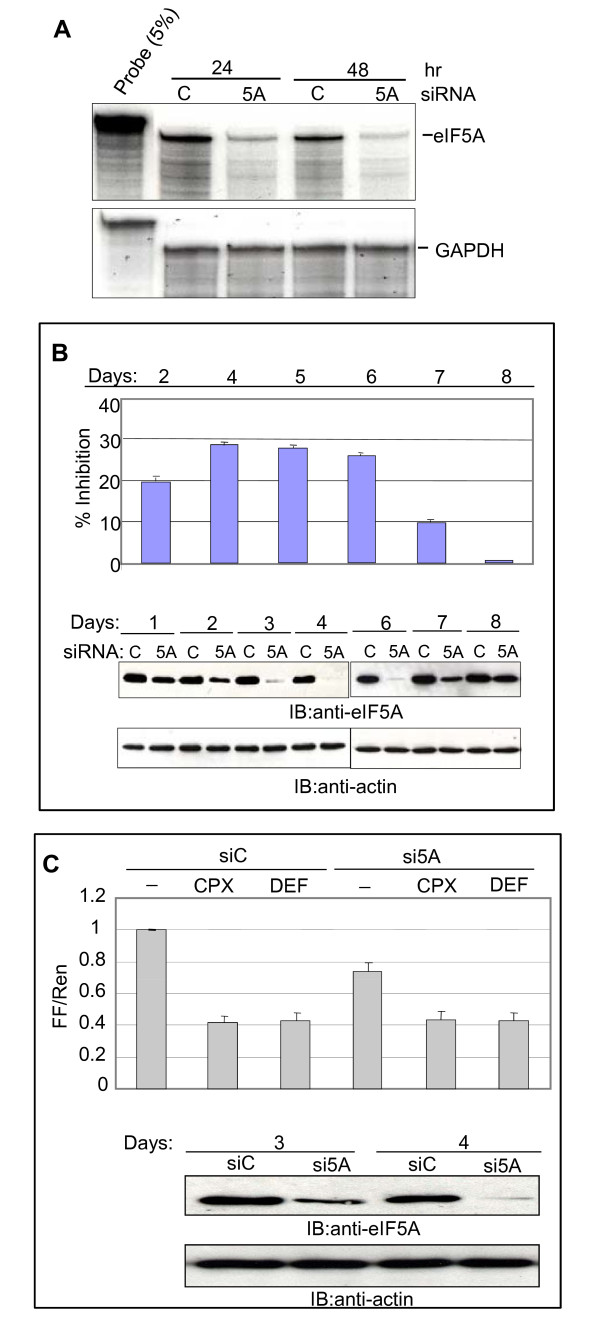
**Depletion of eIF5A by siRNA inhibits gene expression from HIV-1 molecular clone**. **A**. Depletion of eIF5A. 293T cells were transfected with 50 nM of eIF5A-1 siRNA (5A) or control siRNA (C, with no known complementary sequence in the human genome). Total RNA was isolated from transfected cells at the times indicated and analyzed by RPA using probes for eIF5A-1 or GAPDH mRNA (panel A). **B**. Effect of siRNA on HIV gene expression. siRNA-transfected cells were co-transfected with pNL4-3-LucE^- ^and pCMV-Ren at 1, 3, 4, 5, 6 and 7 days after siRNA transfection and harvested 24 hr later for luciferase assays (top panel) as in Fig. 3. Relative FF/Ren luciferase expression at each time point is shown as a percentage inhibition of the control ratio (siC) obtained in the presence of si5A (triplicate measurements ± SD). Parallel cultures were analyzed for eIF5A and actin by immunoblotting (bottom panels). **C**. Lack of synergy between siRNA and drugs. siRNA-transfected 293T cells were additionally transfected with pNL4-3-LucE^- ^and pCMV-Ren 4 days later and simultaneously treated with CPX or DEF as indicated. Luciferase assays were analyzed as in Fig. 3. Immunoblots for eIF5A and actin are shown in the lower panels for days 3 and 4 after siRNA transfection.

The findings described to this point establish a correlation between inhibition of eIF5A modification and inhibition of HIV-1 gene expression. To examine the effect of eIF5A hydroxylation directly we attempted to deplete DOHH by RNA interference. No significant effect on eIF5A modification or HIV gene expression was detected. This is probably because the level of DOHH was not reduced below 60% (data not shown). We therefore turned to siRNA directed against eIF5A-1 itself. Compared to non-targeted control siRNA, eIF5A-1 siRNA reduced the level of its cognate RNA by ~80% at 24 hr (Fig. 7A). The eIF5A protein level declined more gradually, consistent with its long half-life [71], to a minimum of ~30% of control levels at 96 hr post-siRNA transfection (Fig. 7B). GAPDH mRNA and actin protein levels were unchanged, arguing that eIF5A siRNA does not exert a broad deleterious effect in these cells (Fig. 7A, B).

eIF5A knockdown reduced gene expression from the HIV-1 molecular clone by ~30% between 4 and 6 days post-transfection (Fig. 7B, top panel). Although the magnitude of this effect was relatively modest, presumably because of incomplete depletion of eIF5A, two observations attest to its importance. First, the inhibition of HIV-driven gene expression correlated with eIF5A knockdown and recovery (Fig. 7B, lower panel) indicating that targeted reduction of eIF5A expression correlates with inhibition of HIV-driven gene expression. Second, the effects of the drugs and siRNA were not additive. When cells transfected with siRNA for 3 or 4 days were exposed to the drugs for the last 12 hr of this period, eIF5A knockdown did not elicit a further inhibition of HIV-1 gene expression (Fig. 7C). While additional actions cannot be excluded, these observations are consistent with the drugs functioning in the hypusine pathway to inhibit HIV-1 RNA accumulation.

## Discussion

**Figure 8:**
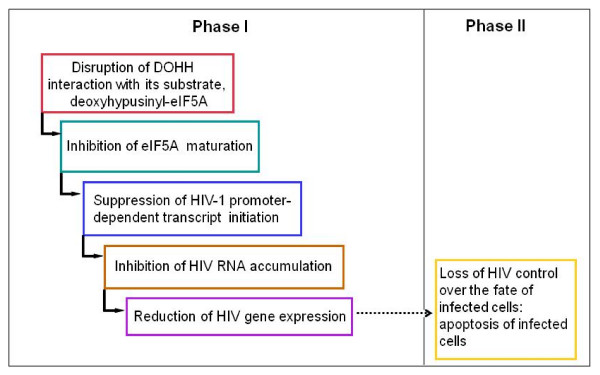
**Scheme for inhibition of HIV-1 infection by CPX and DEF**. The model integrates results presented in this paper for the early phase of drug action with later events leading to apoptosis ([11] Hanauske-Abel et al., in preparation). In phase I the drugs cause a reduction in the expression of HIV proteins, some of which are necessary to prevent apoptosis of HIV infected cells. Apoptosis of infected cells takes place during phase II in the presence of the drugs.

HIV-1 replication can be inhibited by disruption at several different levels of the pathway leading to the post-translational modification of eIF5A with hypusine [10,11,72-75]. The formation of hypusine from lysine requires the sequential action of the enzymes DHS and DOHH. We found that two drugs, CPX and DEF, block eIF5A maturation by inhibiting the interaction between DOHH and its substrate, deoxyhypusyl-eIF5A. At clinically used concentrations, the drugs profoundly inhibited HIV-1 infection in long-term cultures and rapidly reduced HIV-1 gene expression in model systems. CPX and DEF both impaired transcription from the HIV-1 promoter independently of all known viral genes, a mode of action that would be expected to decrease the likelihood of resistance arising during drug treatment. Enhanced susceptibility to apoptosis was reported in an HIV-1 infected cell line treated with DEF [11], and similar findings have been made in PBMCs with both DEF and CPX (Hanauske-Abel et al., in preparation). Hence we propose that inhibition of HIV-1 transcription by these drugs ultimately leads to loss of viral control over the survival of infected cells and to their apoptotic ablation, as outlined in Figure 8.

### CPX and DEF actions

Despite divergent chemical structures, CPX and DEF both act as potent inhibitors of eIF5A maturation in cells and *in vitro *[11,28,41]. DEF is in clinical use as an orally active medicinal chelator for treatment of transfusion-related iron overload, and CPX is employed as a topical antifungal. After oral medication, the concentration of DEF in serum can reach and exceed 250 μM [76]. The topical preparations of CPX, which contain up to 57.5 mM of the agent, achieve levels in excess of 30 μM in skin [77]. Our results were obtained at 250 μM DEF and 30 μM CPX, concentrations well within the range of the drugs' clinically relevant levels. At these concentrations, CPX and DEF can reduce bioavailable intracellular iron levels as determined with an iron-sensitive reporter system, but this effect does not correlate with their antiretroviral action (unpublished data). The medicinal chelator DFOX, which also reduced bioavailable intracellular iron levels, did not inhibit gene expression from the HIV molecular clone (Fig. 3) or block HIV-1 replication when used at clinically relevant levels [60]. This lack of inhibition is consistent with its lack of clinical antiretroviral activity [78]. Several other chelators have been reported to inhibit HIV-1 replication via various possible mechanisms [58,79-82], among them the biologically distinct tridentate drug deferasirox (ICL670) [83]. Agent P2, a bidentate chelation homolog of CPX lacking its hydrophobic cyclohexyl group, displayed little or no activity in our cell-based assays. Thus, the inhibitory action of CPX and DEF on HIV-1 transcription is not merely a consequence of their ability to coordinate and deplete bioavailable iron by bidentate chelation.

CPX and DEF destabilized the interaction between DOHH and deoxyhypusyl-eIF5A, resulting in a marked decrease in the appearance of newly synthesized mature eIF5A (Fig. 2). The drugs did not prevent eIF5A from forming a complex with DHS, which is consistent with the accumulation of deoxyhypusyl-eIF5A in the presence of either drug at concentrations that completely blocked DOHH activity [41]. Neither DFOX nor P2 had any effect on the binding of eIF5A to DOHH, in accordance with their failure to inhibit the formation of hypusinyl-eIF5A. On the other hand, the DHS inhibitor GC7 blocked formation of lysyl-eIF5A:DHS complexes, causing a marked decrease in the levels of deoxyhypusyl-eIF5A and its complexes with DOHH. These findings, supported by molecular modeling (Hanauske-Abel et al., unpublished data), lead us to propose that CPX and DEF enter the deoxyhypusine-binding pocket of DOHH, become oriented towards its catalytic iron atom and chelate it. The drug-iron chelate is then released from the apoenzyme, which irreversible collapses into a catalytically inactive molecule incapable of binding substrate. Supporting this mechanism, DEF is known to cause release of peptide-bound iron from several non-heme metalloproteins, among them mono- and diferric transferrin, cyclooxygenase, and lipoxygenase [84-86].

### Drug effects on HIV-1 gene expression

We analyzed the action of the drugs in a model system consisting of 293T cells transfected with HIV-1 molecular clones. Within 12 hours of their addition, CPX and DEF inhibited gene expression from two different molecular clones, impairing transcription from the viral promoter at the level of initiation. This conclusion is supported by several observations: the inhibition was dependent on the HIV-1 5'-LTR. No specific effects were detectable on transcription elongation or on downstream mRNA processing, transport or stability. Gene expression from another promoter (the CMV immediate early promoter) and the levels of cellular proteins (actin, eIF5A) were unaffected by CPX and DEF. It is notable that hydroxyurea, which has clinically relevant antiretroviral activity [87,88], also rapidly inhibits transactivation of the HIV promoter to a similar degree [89]. While the concentrations of hydroxyurea required exceed those of CPX by almost two orders of magnitude, the presence of a hydroxyurea-like domain in the CPX structure may imply a common mechanism of action.

eIF5A has also been reported to play post-transcriptional roles in HIV gene expression. The work of Hauber and colleagues identified eIF5A as a cellular cofactor for Rev [7,10-13], leading to the expectation that CPX and DEF would block the export of under-spliced HIV-1 RNAs from the nucleus in a Rev-dependent manner. This prediction was not substantiated in our experiments, however. First, the sensitivity of FF expression from pNL4-3-LucE^- ^to the drugs (Fig. 3) implies a Rev-independent action because the FF luciferase gene is inserted into the HIV-1 *nef *gene. The HIV-1 *nef *gene's mRNA is fully spliced and transported independently of Rev. Second, the drugs reduced the accumulation of unspliced and spliced RNA from pNL4-3-LucE^- ^to approximately equal extents and the decrease in RNA accumulation occurred in both nuclear and cytoplasmic compartments (Fig. 4). Third, the drugs inhibited RNA expression from the *rev*-minus molecular clone pMRev(-) (Fig. 4), and from several deletion constructs that lack *rev *(Fig. 5). We therefore conclude that Rev is not involved in the effects of CPX and DEF reported here. Similarly, CPX and DEF inhibited the expression of p24, which is translated from incompletely spliced *gag *mRNA, to about the same extent as FF, generated from spliced mRNA (Fig. 3D). In another study, DEF and mimosine (a naturally occurring analog) were shown to reduce the association of *gag *mRNA with polysomes, implying an effect on p24 synthesis at the translational level [11]. While this action could be compatible with an eIF5A role in protein synthesis [16-19], it does not comport with the inhibition of transcription documented here. Sharp differences between the experimental systems employed mitigate against direct comparisons. The findings of Andrus and co-workers were made using latently infected ACH-2 cells induced by a phorbol ester [11], which entails multiple effects including sequelae of PKC activation. Moreover, the experiments were conducted over a longer term than those reported here, increasing the opportunity for additional effects to be manifested. The short time span of our experiments was designed to uncover the primary effects of the drugs on HIV-1 gene expression. Nevertheless, it cannot be ruled out that eIF5A also affects other processes and these effects may become more evident with longer drug exposure.

### Role of eIF5A

eIF5A-1 depletion by siRNA reduced HIV-driven gene expression in a manner that was not additive with the action of CPX and DEF. DOHH knockdown by siRNA did not significantly impair HIV gene expression in 293T cells, but expression from the viral promoter was reduced by ~50% in HeLa cells (data not shown). Knockdown of DHS or of eIF5A-1 in HeLa cells elicited similar effects. Although other drug actions (including the inhibition of other hydroxylases) are not excluded, these findings strengthen the view that the sensitivity of HIV-1 to CPX and DEF results at least in part from their action on eIF5A maturation. How this affects transcription initiation remains to be elucidated. Despite a large body of literature documenting the function of eIF5A at various levels of gene expression, its mechanism(s) of action remain elusive. Interestingly, connections exist between eIF5A and HIV transcription via the cell cycle: hypusine-containing eIF5A is required for G1/S passage as noted above, and HIV-1 is transcribed efficiently in the G2 phase [90-92] although not exclusively so [93]. Thus, inhibition of eIF5A modification could affect both HIV transcription and the cell cycle through eIF5A-dependant cellular components that are common to both pathways. Further work will be required to define the function of mature eIF5A in HIV-1 infection and to establish the sequence of molecular events engendered by CPX and DEF. Our findings provide a mechanistic rationale to study such drugs for their ability to suppress HIV-1 infection in patients, and encourage the development of antiretroviral agents that target the posttranslational formation of hypusine in eIF5A.

## Conclusion

Ciclopirox and deferiprone, two clinically used drugs, block HIV-1 infection. In model systems, the drugs inhibit the enzyme DOHH required for maturation of eIF5A and repress expression from the HIV-1 promoter at the level of transcription initiation. Our results support the concept that drugs targeting DOHH should be tested clinically for HIV-1 inhibition and could be developed as antiretrovirals.

## Materials and methods

### Reagents

Deferiprone was purchased from Calbiochem. Ciclopirox, deferoxamine and actinomycin D were purchased from Sigma. Agent P2 was synthesized and characterized as described by Hanauske-Abel et al. (in preparation). Rabbit NIH-353 antibody was raised against mature human eIF5A [35]. Antibody against DOHH was generated by M. H. Park. Anti-eIF5A-1 monoclonal antibody (BD) was purchased from BD Biosciences. The anti-FLAG monoclonal antibody M2 and anti-actin antibody were purchased from Sigma.

### Cells

293T and COS7 cells were grown in DME medium (Sigma) and Jurkat cells in RPMI medium (Sigma), both supplemented with penicillin, streptomycin and 8% FBS. Quantitation of apoptosis and viability was performed with a BD FACSCalibur™ system using Annexin V-PE Apoptosis Detection Kit I (BD Biosciences, San Jose CA).

### Plasmids

pSP-luc+ and pSP-rluc were purchased from Promega, Madison. The HIV-1 molecular clone pMRev(-), and the Rev expression vector, plasmid pCMV-Rev, were obtained from the NIH AIDS Research and Reference Reagent Program. FLAG-tagged Rev and eIF5A expression vectors were made by sub-cloning Rev and eIF5A sequences respectively, into the pcDNA3.1FLAG vector. pBSII-HIV+80-340 was constructed by subcloning PCR-amplified HIV-1 sequence (+80-340) from pNL4-3-LucE^- ^[94] into the pBSIIKS+ Bluescript vector. pNL4-3-LucE^- ^truncations were generated by deleting sequences using suitable restriction enzymes. Truncation III was made by deleting sequence from nt 1506 to 5784 using SpeI and SalI enzymes. Similarly, truncations IV (nt 5784 to 8464) and V (nt 712 to 8464) were made with SaII and BamHI and with BssHII and BamHI, respectively. The plasmid pGL2TAR was obtained from Dr. David Price and contains most of the HIV-1 LTR (from KpnI to HindIII). To generate construct VI, the HindIII to PflM1 sequence from pGL2TAR was replaced by the HindIII to XhoI sequence from construct V. Construct VII (pLTR-FF) was made by substituting the sequence between the ClaI and BsgI sites of pGL2TAR with the ClaI to BsgI fragment from construct VI.

### HIV-1 infection of PBMC cultures

Uninfected PBMCs from a healthy donor were incubated with infected PBMCs at a 10:1 ratio (1 × 10^6 ^cells) in 24-well microplates in RPMI medium with antibiotics, glutamate, IL-2 and 10% fetal calf serum. CPX, DEF, or agent P2 was added 48 hr later. Infected PBMCs were isolated from a highly immunocompromised donor (#990,135: CD4 count < 5%; HIV RNA in plasma at log_10 _5.5 copies/ml) using an IRB-approved protocol. Half of the medium supplemented with the appropriate compound was replenished every 24 hr leaving the cell layer undisturbed. A set of wells was harvested every 24 hr for the p24 assay.

### Transfection and luciferase assays

Plasmids were introduced into 2 × 10^5 ^293T cells by transfection using Lipofectamine 2000 (Invitrogen) according to the manufacturer's instructions. Compounds (such as CPX or DEF) were added simultaneously. Cells were harvested at 12 hr post-transfection, washed with PBS, lysed in 0.15 ml of 1× passive lysis buffer (Promega), and assayed for luciferase activity using the Promega dual luciferase reporter system according to the manufacturer's instructions. Jurkat cells (1 × 10^6 ^cells) were transfected using FuGENE 6 (Roche) according to the manufacturer's instructions and assayed in a similar fashion after pelleting.

### Quantitation of p24

p24 core antigen was quantified in PBMC culture supernatants and transfected cell extracts using a commercially available ELISA (Retrotek HIV-1 p24™; ZeptoMetrix Corp.; Buffalo, NY).

### Preparation of nuclear and cytoplasmic RNA

293T cells (2 × 10^6 ^cells) were seeded in 10-cm-diameter plates and transfected 20 hr later by using Transfectene (Bio-Rad) and treated with compounds. Cells were harvested at 15 hr post-transfection and suspended in a low salt buffer (10 mM Tris. HCl pH 7.4, 10 mM NaCl, 1.5 mM MgCl_2_, and 0.5% NP-40). Cells were vortexed for 10 sec and incubated on ice for 10 min. Cell extracts were centrifuged at 500 × g for 3 min, followed by cytoplasmic and nuclear RNA isolation from the supernatant and the pellet, respectively, using Trizol (Invitrogen) according to the manufacturer's instructions.

### RNase protection assay (RPA)

RPA was performed with 10 μg of cytoplasmic RNA and 5 μg of nuclear RNA, using the RPAIII kit from Ambion (Austin, TX) according to the manufacturer's instructions. Synthesis of radiolabeled RNA and protection assays were performed as described previously [95]. To generate antisense RNA probe against the HIV-1 major splice site, firefly luciferase and *Renilla *luciferase pBSII-KS+HIV (+80-341), pSP-luc and pSP-rluc were linearized with HindIII, XbaI and BsaI, respectively. The resulting probes were 309, 390 and 245 nt long, respectively. The antisense HIV-1 leader RNA probe complementary to nt +83 to -117 of the LTR was generated by subcloning between the XbaI and HindIII sites of the pcDNA3.1 vector. Antisense probe corresponding to the N terminus of eIF5A was generated by subcloning 250 nt of its cDNA sequence into pcDNA3.1.

### Immunoprecipitation and immunoblotting

Immunoprecipitation and immunoblotting experiments were carried out as described previously [96].

### RNA interference

A pool of four siRNAs targeting eIF5A-1 mRNA (ON-TARGET *plus SMART*pool^®^), sequence-specific siRNA against DOHH, and control siRNA were purchased from Dharmacon Inc. Cells were transfected with 50 nM siRNA using HiPerFect transfection reagent (Qiagen) according to the manufacturer's instructions. The effectiveness of siRNA against specific targets was determined by RPA and immunoblotting.

## Competing interests

The authors declare that they have no competing interests.

## Authors' contributions

MH designed and conducted the experiments, analyzed the data, generated the figures, and participated in writing the manuscript. HMHA, PP and MBM initiated the project. TP and MBM designed the experiments, analyzed the data, and wrote the manuscript. HMHA analyzed the data and participated in writing the manuscript. DDGA and MHP designed and contributed reagents. MHP, PP and DS provided conceptual input. All authors have read and approved the final manuscript.

## References

[B1] Lagakos SW, Gable AR (2008). Challenges to HIV prevention--seeking effective measures in the absence of a vaccine. N Engl J Med.

[B2] Emerman M, Malim MH (1998). HIV-1 regulatory/accessory genes: keys to unraveling viral and host cell biology. Science.

[B3] Baba M (2006). Recent status of HIV-1 gene expression inhibitors. Antiviral Res.

[B4] Lau A, Swinbank KM, Ahmed PS, Taylor DL, Jackson SP, Smith GC, O'Connor MJ (2005). Suppression of HIV-1 infection by a small molecule inhibitor of the ATM kinase. Nat Cell Biol.

[B5] Jenkins ZA, Haag PG, Johansson HE (2001). Human eIF5A2 on chromosome 3q25-q27 is a phylogenetically conserved vertebrate variant of eukaryotic translation initiation factor 5A with tissue-specific expression. Genomics.

[B6] Clement PM, Henderson CA, Jenkins ZA, Smit-McBride Z, Wolff EC, Hershey JW, Park MH, Johansson HE (2003). Identification and characterization of eukaryotic initiation factor 5A-2. Eur J Biochem.

[B7] Ruhl M, Himmelspach M, Bahr GM, Hammerschmid F, Jaksche H, Wolff B, Aschauer H, Farrington GK, Probst H, Bevec D (1993). Eukaryotic initiation factor 5A is a cellular target of the human immunodeficiency virus type 1 Rev activation domain mediating trans-activation. J Cell Biol.

[B8] Bevec D, Jaksche H, Oft M, Wohl T, Himmelspach M, Pacher A, Schebesta M, Koettnitz K, Dobrovnik M, Csonga R (1996). Inhibition of HIV-1 replication in lymphocytes by mutants of the Rev cofactor eIF-5A. Science.

[B9] Junker U, Bevec D, Barske C, Kalfoglou C, Escaich S, Dobrovnik M, Hauber J, Bohnlein E (1996). Intracellular expression of cellular eIF-5A mutants inhibits HIV-1 replication in human T cells: a feasibility study. Hum Gene Ther.

[B10] Hauber I, Bevec D, Heukeshoven J, Kratzer F, Horn F, Choidas A, Harrer T, Hauber J (2005). Identification of cellular deoxyhypusine synthase as a novel target for antiretroviral therapy. J Clin Invest.

[B11] Andrus L, Szabo P, Grady RW, Hanauske AR, Huima-Byron T, Slowinska B, Zagulska S, Hanauske-Abel HM (1998). Antiretroviral effects of deoxyhypusyl hydroxylase inhibitors: a hypusine-dependent host cell mechanism for replication of human immunodeficiency virus type 1 (HIV-1). Biochem Pharmacol.

[B12] Bevec D, Hauber J (1997). Eukaryotic initiation factor 5A activity and HIV-1 Rev function. Biol Signals.

[B13] Rosorius O, Reichart B, Kratzer F, Heger P, Dabauvalle MC, Hauber J (1999). Nuclear pore localization and nucleocytoplasmic transport of eIF-5A: evidence for direct interaction with the export receptor CRM1. J Cell Sci.

[B14] Elfgang C, Rosorius O, Hofer L, Jaksche H, Hauber J, Bevec D (1999). Evidence for specific nucleocytoplasmic transport pathways used by leucine-rich nuclear export signals. Proc Natl Acad Sci USA.

[B15] Hofmann W, Reichart B, Ewald A, Muller E, Schmitt I, Stauber RH, Lottspeich F, Jockusch BM, Scheer U, Hauber J, Dabauvalle MC (2001). Cofactor requirements for nuclear export of Rev response element (RRE)- and constitutive transport element (CTE)-containing retroviral RNAs. An unexpected role for actin. J Cell Biol.

[B16] Zanelli CF, Valentini SR (2007). Is there a role for eIF5A in translation?. Amino Acids.

[B17] Zanelli CF, Maragno AL, Gregio AP, Komili S, Pandolfi JR, Mestriner CA, Lustri WR, Valentini SR (2006). eIF5A binds to translational machinery components and affects translation in yeast. Biochem Biophys Res Commun.

[B18] Jao DL, Chen KY (2006). Tandem affinity purification revealed the hypusine-dependent binding of eukaryotic initiation factor 5A to the translating 80S ribosomal complex. J Cell Biochem.

[B19] Saini P, Eyler DE, Green R, Dever TE (2009). Hypusine-containing protein eIF5A promotes translation elongation. Nature.

[B20] Lipowsky G, Bischoff FR, Schwarzmaier P, Kraft R, Kostka S, Hartmann E, Kutay U, Gorlich D (2000). Exportin 4: a mediator of a novel nuclear export pathway in higher eukaryotes. Embo J.

[B21] Zuk D, Jacobson A (1998). A single amino acid substitution in yeast eIF-5A results in mRNA stabilization. Embo J.

[B22] Schrader R, Young C, Kozian D, Hoffmann R, Lottspeich F (2006). Temperature-sensitive eIF5A mutant accumulates transcripts targeted to the nonsense-mediated decay pathway. J Biol Chem.

[B23] Benne R, Brown-Luedi ML, Hershey JW (1978). Purification and characterization of protein synthesis initiation factors eIF-1, eIF-4C, eIF-4D, and eIF-5 from rabbit reticulocytes. J Biol Chem.

[B24] Cooper HL, Park MH, Folk JE, Safer B, Braverman R (1983). Identification of the hypusine-containing protein hy+ as translation initiation factor eIF-4D. Proc Natl Acad Sci USA.

[B25] Liu YP, Nemeroff M, Yan YP, Chen KY (1997). Interaction of eukaryotic initiation factor 5A with the human immunodeficiency virus type 1 Rev response element RNA and U6 snRNA requires deoxyhypusine or hypusine modification. Biol Signals.

[B26] Xu A, Chen KY (2001). Hypusine is required for a sequence-specific interaction of eukaryotic initiation factor 5A with postsystematic evolution of ligands by exponential enrichment RNA. J Biol Chem.

[B27] Kang HA, Hershey JW (1994). Effect of initiation factor eIF-5A depletion on protein synthesis and proliferation of Saccharomyces cerevisiae. J Biol Chem.

[B28] Hanauske-Abel HM, Slowinska B, Zagulska S, Wilson RC, Staiano-Coico L, Hanauske AR, McCaffrey T, Szabo P (1995). Detection of a sub-set of polysomal mRNAs associated with modulation of hypusine formation at the G1-S boundary. Proposal of a role for eIF-5A in onset of DNA replication. FEBS Lett.

[B29] Taylor CA, Sun Z, Cliche DO, Ming H, Eshaque B, Jin S, Hopkins MT, Thai B, Thompson JE (2007). Eukaryotic translation initiation factor 5A induces apoptosis in colon cancer cells and associates with the nucleus in response to tumour necrosis factor alpha signalling. Exp Cell Res.

[B30] Clement PM, Johansson HE, Wolff EC, Park MH (2006). Differential expression of eIF5A-1 and eIF5A-2 in human cancer cells. Febs J.

[B31] Caraglia M, Marra M, Giuberti G, D'Alessandro AM, Budillon A, del Prete S, Lentini A, Beninati S, Abbruzzese A (2001). The role of eukaryotic initiation factor 5A in the control of cell proliferation and apoptosis. Amino Acids.

[B32] Wei L, Wang Z, Cui T, Yi F, Bu Y, Ding S, Ma Y, Song F (2007). Proteomic Analysis of Cervical Cancer Cells Treated with Adenovirus-Mediated MDA-7. Cancer Biol Ther.

[B33] Huang Y, Higginson DS, Hester L, Park MH, Snyder SH (2007). Neuronal growth and survival mediated by eIF5A, a polyamine-modified translation initiation factor. Proc Natl Acad Sci USA.

[B34] Chatterjee I, Gross SR, Kinzy TG, Chen KY (2006). Rapid depletion of mutant eukaryotic initiation factor 5A at restrictive temperature reveals connections to actin cytoskeleton and cell cycle progression. Mol Genet Genomics.

[B35] Cracchiolo BM, Heller DS, Clement PM, Wolff EC, Park MH, Hanauske-Abel HM (2004). Eukaryotic initiation factor 5A-1 (eIF5A-1) as a diagnostic marker for aberrant proliferation in intraepithelial neoplasia of the vulva. Gynecol Oncol.

[B36] Guan XY, Sham JS, Tang TC, Fang Y, Huo KK, Yang JM (2001). Isolation of a novel candidate oncogene within a frequently amplified region at 3q26 in ovarian cancer. Cancer Res.

[B37] Guan XY, Fung JM, Ma NF, Lau SH, Tai LS, Xie D, Zhang Y, Hu L, Wu QL, Fang Y, Sham JS (2004). Oncogenic role of eIF-5A2 in the development of ovarian cancer. Cancer Res.

[B38] Schnier J, Schwelberger HG, Smit-McBride Z, Kang HA, Hershey JW (1991). Translation initiation factor 5A and its hypusine modification are essential for cell viability in the yeast Saccharomyces cerevisiae. Mol Cell Biol.

[B39] Cano VS, Jeon GA, Johansson HE, Henderson CA, Park JH, Valentini SR, Hershey JW, Park MH (2008). Mutational analyses of human eIF5A-1--identification of amino acid residues critical for eIF5A activity and hypusine modification. Febs J.

[B40] Hanauske-Abel HM, Park MH, Hanauske AR, Popowicz AM, Lalande M, Folk JE (1994). Inhibition of the G1-S transition of the cell cycle by inhibitors of deoxyhypusine hydroxylation. Biochim Biophys Acta.

[B41] Clement PM, Hanauske-Abel HM, Wolff EC, Kleinman HK, Park MH (2002). The antifungal drug ciclopirox inhibits deoxyhypusine and proline hydroxylation, endothelial cell growth and angiogenesis in vitro. Int J Cancer.

[B42] Lee Y, Kim HK, Park HE, Park MH, Joe YA (2002). Effect of N1-guanyl-1,7-diaminoheptane, an inhibitor of deoxyhypusine synthase, on endothelial cell growth, differentiation and apoptosis. Mol Cell Biochem.

[B43] Wang G, Miskimins R, Miskimins WK (2000). Mimosine arrests cells in G1 by enhancing the levels of p27(Kip1). Exp Cell Res.

[B44] Balabanov S, Gontarewicz A, Ziegler P, Hartmann U, Kammer W, Copland M, Brassat U, Priemer M, Hauber I, Wilhelm T (2007). Hypusination of eukaryotic initiation factor 5A (eIF5A): a novel therapeutic target in BCR-ABL-positive leukemias identified by a proteomics approach. Blood.

[B45] Klier H, Csonga R, Joao HC, Eckerskorn C, Auer M, Lottspeich F, Eder J (1995). Isolation and structural characterization of different isoforms of the hypusine-containing protein eIF-5A from HeLa cells. Biochemistry.

[B46] Shirai A, Matsuyama A, Yashiroda Y, Hashimoto A, Kawamura Y, Arai R, Komatsu Y, Horinouchi S, Yoshida M (2008). Global analysis of gel mobility of proteins and its use in target identification. J Biol Chem.

[B47] Park MH (2006). The post-translational synthesis of a polyamine-derived amino acid, hypusine, in the eukaryotic translation initiation factor 5A (eIF5A). J Biochem.

[B48] Chattopadhyay MK, Park MH, Tabor H (2008). Hypusine modification for growth is the major function of spermidine in Saccharomyces cerevisiae polyamine auxotrophs grown in limiting spermidine. Proc Natl Acad Sci USA.

[B49] Wolff EC, Kang KR, Kim YS, Park MH (2007). Posttranslational synthesis of hypusine: evolutionary progression and specificity of the hypusine modification. Amino Acids.

[B50] Dou QP, Chen KY (1990). Characterization and reconstitution of a cell free system for NAD(+)-dependent deoxyhypusine formation on the 18 kDa eIF-4D precursor. Biochim Biophys Acta.

[B51] Lee YH, Koh SS, Zhang X, Cheng X, Stallcup MR (2002). Synergy among nuclear receptor coactivators: selective requirement for protein methyltransferase and acetyltransferase activities. Mol Cell Biol.

[B52] Park JH, Aravind L, Wolff EC, Kaevel J, Kim YS, Park MH (2006). Molecular cloning, expression, and structural prediction of deoxyhypusine hydroxylase: a HEAT-repeat-containing metalloenzyme. Proc Natl Acad Sci USA.

[B53] Patel PH, Costa-Mattioli M, Schulze KL, Bellen HJ (2009). The Drosophila deoxyhypusine hydroxylase homologue nero and its target eIF5A are required for cell growth and the regulation of autophagy. J Cell Biol.

[B54] Abbruzzese A, Hanauske-Abel HM, Park MH, Henke S, Folk JE (1991). The active site of deoxyhypusyl hydroxylase: use of catecholpeptides and their component chelator and peptide moieties as molecular probes. Biochim Biophys Acta.

[B55] Csonga R, Ettmayer P, Auer M, Eckerskorn C, Eder J, Klier H (1996). Evaluation of the metal ion requirement of the human deoxyhypusine hydroxylase from HeLa cells using a novel enzyme assay. FEBS Lett.

[B56] Gupta AK, Plott T (2004). Ciclopirox: a broad-spectrum antifungal with antibacterial and anti-inflammatory properties. Int J Dermatol.

[B57] Chan JC, Chim CS, Ooi CG, Cheung B, Liang R, Chan TK, Chan V (2006). Use of the oral chelator deferiprone in the treatment of iron overload in patients with Hb H disease. Br J Haematol.

[B58] Georgiou NA, van der Bruggen T, Oudshoorn M, Nottet HS, Marx JJ, van Asbeck BS (2000). Inhibition of human immunodeficiency virus type 1 replication in human mononuclear blood cells by the iron chelators deferoxamine, deferiprone, and bleomycin. J Infect Dis.

[B59] Choudhry VP, Naithani R (2007). Current status of iron overload and chelation with deferasirox. Indian J Pediatr.

[B60] Lazdins JK, Alteri E, Klimkait T, Woods-Cook K, Walker MR, Goutte G, Poncioni B (1991). Lack of effect of desferrioxamine on in-vitro HIV-1 replication. Lancet.

[B61] Lee YB, Joe YA, Wolff EC, Dimitriadis EK, Park MH (1999). Complex formation between deoxyhypusine synthase and its protein substrate, the eukaryotic translation initiation factor 5A (eIF5A) precursor. Biochem J.

[B62] Thompson GM, Cano VS, Valentini SR (2003). Mapping eIF5A binding sites for Dys1 and Lia1: in vivo evidence for regulation of eIF5A hypusination. FEBS Lett.

[B63] Kang KR, Kim YS, Wolff EC, Park MH (2007). Specificity of the deoxyhypusine hydroxylase-eukaryotic translation initiation factor (eIF5A) interaction: identification of amino acid residues of the enzyme required for binding of its substrate, deoxyhypusine-containing eIF5A. J Biol Chem.

[B64] Jakus J, Wolff EC, Park MH, Folk JE (1993). Features of the spermidine-binding site of deoxyhypusine synthase as derived from inhibition studies. Effective inhibition by bis- and mono-guanylated diamines and polyamines. J Biol Chem.

[B65] Umland TC, Wolff EC, Park MH, Davies DR (2004). A new crystal structure of deoxyhypusine synthase reveals the configuration of the active enzyme and of an enzyme.NAD.inhibitor ternary complex. J Biol Chem.

[B66] Zheng YH, Yu HF, Peterlin BM (2003). Human p32 protein relieves a post-transcriptional block to HIV replication in murine cells. Nat Cell Biol.

[B67] Sadaie MR, Benter T, Wong-Staal F (1988). Site-directed mutagenesis of two trans-regulatory genes (tat-III, trs) of HIV-1. Science.

[B68] Zhu Y, Pe'ery T, Peng J, Ramanathan Y, Marshall N, Marshall T, Amendt B, Mathews MB, Price DH (1997). Transcription elongation factor P-TEFb is required for HIV-1 tat transactivation in vitro. Genes Dev.

[B69] Kao SY, Calman AF, Luciw PA, Peterlin BM (1987). Anti-termination of transcription within the long terminal repeat of HIV-1 by tat gene product. Nature (London).

[B70] Laspia MF, Rice AP, Mathews MB (1989). HIV-1 Tat protein increases transcriptional initiation and stabilizes elongation. Cell.

[B71] Nishimura K, Murozumi K, Shirahata A, Park MH, Kashiwagi K, Igarashi K (2005). Independent roles of eIF5A and polyamines in cell proliferation. Biochem J.

[B72] Schafer B, Hauber I, Bunk A, Heukeshoven J, Dusedau A, Bevec D, Hauber J (2006). Inhibition of multidrug-resistant HIV-1 by interference with cellular S-adenosylmethionine decarboxylase activity. J Infect Dis.

[B73] Chiang PK, McCann PP, Lane JR, Pankaskie MC, Burke DS, Mayers DL (1996). Antihuman Immunodeficiency Virus (HIV-1) Activities of Inhibitors of Polyamine Pathways. J Biomed Sci.

[B74] Marasco CJ, Kramer DL, Miller J, Porter CW, Bacchi CJ, Rattendi D, Kucera L, Iyer N, Bernacki R, Pera P, Sufrin JR (2002). Synthesis and evaluation of analogues of 5'-([(Z)-4-amino-2-butenyl]methylamino)-5'-deoxyadenosine as inhibitors of tumor cell growth, trypanosomal growth, and HIV-1 infectivity. J Med Chem.

[B75] Hart RA, Billaud JN, Choi SJ, Phillips TR (2002). Effects of 1,8-diaminooctane on the FIV Rev regulatory system. Virology.

[B76] Kontoghiorghes GJ, Goddard JG, Bartlett AN, Sheppard L (1990). Pharmacokinetic studies in humans with the oral iron chelator 1,2-dimethyl-3-hydroxypyrid-4-one. Clin Pharmacol Ther.

[B77] Gupta AK (2001). Ciclopirox: an overview. Int J Dermatol.

[B78] Salhi Y, Costagliola D, Rebulla P, Dessi C, Karagiorga M, Lena-Russo D, de Montalembert M, Girot R (1998). Serum ferritin, desferrioxamine, and evolution of HIV-1 infection in thalassemic patients. J Acquir Immune Defic Syndr Hum Retrovirol.

[B79] Debebe Z, Ammosova T, Jerebtsova M, Kurantsin-Mills J, Niu X, Charles S, Richardson DR, Ray PE, Gordeuk VR, Nekhai S (2007). Iron chelators ICL670 and 311 inhibit HIV-1 transcription. Virology.

[B80] Sappey C, Boelaert JR, Legrand-Poels S, Forceille C, Favier A, Piette J (1995). Iron chelation decreases NF-kappa B and HIV type 1 activation due to oxidative stress. AIDS Res Hum Retroviruses.

[B81] van Asbeck BS, Georgiou NA, Bruggen T van der, Oudshoorn M, Nottet HS, Marx JJ (2001). Anti-HIV effect of iron chelators: different mechanisms involved. J Clin Virol.

[B82] Georgiou NA, Bruggen T van der, Oudshoorn M, Hider RC, Marx JJ, van Asbeck BS (2002). Human immunodeficiency virus type 1 replication inhibition by the bidentate iron chelators CP502 and CP511 is caused by proliferation inhibition and the onset of apoptosis. Eur J Clin Invest.

[B83] Nick H, Acklin P, Lattmann R, Buehlmayer P, Hauffe S, Schupp J, Alberti D (2003). Development of tridentate iron chelators: from desferrithiocin to ICL670. Curr Med Chem.

[B84] Li Y, Harris WR (1998). Iron removal from monoferric human serum transferrins by 1, 2-dimethyl-3-hydroxypyridin-4-one, 1-hydroxypyridin-2-one and acetohydroxamic acid. Biochim Biophys Acta.

[B85] Barradas MA, Jeremy JY, Kontoghiorghes GJ, Mikhailidis DP, Hoffbrand AV, Dandona P (1989). Iron chelators inhibit human platelet aggregation, thromboxane A2 synthesis and lipoxygenase activity. FEBS Lett.

[B86] Kontoghiorghes GJ, Evans RW (1985). Site specificity of iron removal from transferrin by alpha-ketohydroxypyridine chelators. FEBS Lett.

[B87] Lisziewicz J, Foli A, Wainberg M, Lori F (2003). Hydroxyurea in the treatment of HIV infection: clinical efficacy and safety concerns. Drug Saf.

[B88] Garcia F, Plana M, Arnedo M, Ortiz GM, Miro JM, Lopalco L, Lori F, Pumarola T, Gallart T, Gatell JM (2003). A cytostatic drug improves control of HIV-1 replication during structured treatment interruptions: a randomized study. AIDS.

[B89] Calzado MA, MacHo A, Lucena C, Munoz E (2000). Hydroxyurea inhibits the transactivation of the HIV-long-terminal repeat (LTR) promoter. Clin Exp Immunol.

[B90] Gummuluru S, Emerman M (1999). Cell cycle- and Vpr-mediated regulation of human immunodeficiency virus type 1 expression in primary and transformed T-cell lines. J Virol.

[B91] Wang D, de la Fuente C, Deng L, Wang L, Zilberman I, Eadie C, Healey M, Stein D, Denny T, Harrison LE (2001). Inhibition of human immunodeficiency virus type 1 transcription by chemical cyclin-dependent kinase inhibitors. J Virol.

[B92] Thierry S, Marechal V, Rosenzwajg M, Sabbah M, Redeuilh G, Nicolas JC, Gozlan J (2004). Cell cycle arrest in G2 induces human immunodeficiency virus type 1 transcriptional activation through histone acetylation and recruitment of CBP, NF-kappaB, and c-Jun to the long terminal repeat promoter. J Virol.

[B93] Kundu M, Sharma S, De Luca A, Giordano A, Rappaport J, Khalili K, Amini S (1998). HIV-1 Tat elongates the G1 phase and indirectly promotes HIV-1 gene expression in cells of glial origin. J Biol Chem.

[B94] Chen BK, Saksela K, Andino R, Baltimore D (1994). Distinct modes of human immunodeficiency virus type 1 proviral latency revealed by superinfection of nonproductively infected cell lines with recombinant luciferase-encoding viruses. J Virol.

[B95] Young TM, Wang Q, Pe'ery T, Mathews MB (2003). The Human I-mfa Domain-Containing Protein, HIC, Interacts with Cyclin T1 and Modulates P-TEFb-Dependent Transcription. Mol Cell Biol.

[B96] Hoque M, Young TM, Lee CG, Serrero G, Mathews MB, Pe'ery T (2003). The Growth Factor Granulin Interacts with Cyclin T1 and Modulates P-TEFb-Defendent Transcription. Mol Cell Biol.

